# Fabrication of Microfluidic Devices for Continuously Monitoring Yeast Aging

**DOI:** 10.21769/BioProtoc.4782

**Published:** 2023-08-05

**Authors:** Richard O’Laughlin, Emerald Forrest, Jeff Hasty, Nan Hao

**Affiliations:** 1 Department of Bioengineering, University of California San Diego, La Jolla, CA 92093, USA; 2 Synthetic Biology Institute, University of California, San Diego, La Jolla, CA 92093, USA; 3 Department of Molecular Biology, School of Biological Sciences, University of California San Diego, La Jolla, CA 92093, USA

**Keywords:** Aging, Yeast replicative aging, *Saccharomyces cerevisiae*, Microfluidics, Microfabrication, Photolithography, Soft lithography, Polydimethylsiloxane (PDMS)

## Abstract

For several decades, aging in *Saccharomyces cerevisiae* has been studied in hopes of understanding its causes and identifying conserved pathways that also drive aging in multicellular eukaryotes. While the short lifespan and unicellular nature of budding yeast has allowed its aging process to be observed by dissecting mother cells away from daughter cells under a microscope, this technique does not allow continuous, high-resolution, and high-throughput studies to be performed. Here, we present a protocol for constructing microfluidic devices for studying yeast aging that are free from these limitations. Our approach uses multilayer photolithography and soft lithography with polydimethylsiloxane (PDMS) to construct microfluidic devices with distinct single-cell trapping regions as well as channels for supplying media and removing recently born daughter cells. By doing so, aging yeast cells can be imaged at scale for the entirety of their lifespans, and the dynamics of molecular processes within single cells can be simultaneously tracked using fluorescence microscopy.

Key features

This protocol requires access to a photolithography lab in a cleanroom facility.

Photolithography process for patterning photoresist on silicon wafers with multiple different feature heights.

Soft lithography process for making PDMS microfluidic devices from silicon wafer templates.

## Background

Progress in aging research has been greatly accelerated in recent years due to the development of new tools and techniques for single-cell analysis (Dulken et al., 2019; Li et al., 2020; Tabula Muris, 2020; Trapp et al., 2021; Roux et al., 2022). Microfluidic technologies play pivotal roles in these applications, as they allow single cells to be captured in droplets for sequencing (Matuła et al., 2020) or isolated for long-term imaging (Allard et al., 2022). For studying replicative aging in *Saccharomyces cerevisiae*, the traditional method of manual microdissection of mother and daughter cells in order to count replicative lifespan has been largely supplanted by the use of microfluidic devices, which efficiently trap individual mother cells in place while removing newly budded daughter cells [reviewed in Chen et al. (2017); Gao et al. (2020); O’Laughlin et al. (2020)]. Since mother cells growing in microfluidic devices can be imaged at regular intervals, and various molecular and biochemical processes can be monitored in real-time with fluorescence microscopy, this approach has led to a number of significant advances in understanding yeast aging (O’Laughlin et al., 2020). Prominent among these is the discovery of two divergent trajectories that single cells take as they age, which are marked by distinct morphological features and the breakdown of different cellular functions (Li et al., 2017 and 2020; Jin et al., 2019; Paxman et al., 2022). Recently, our group has used these devices to reveal that cells undergoing one of the aging trajectories display a reduction in protein homeostasis, with RNA binding proteins aggregating after diminished control of chromatin silencing at the rDNA locus (Paxman et al., 2022). Here, we report a detailed protocol for constructing the microfluidic devices for yeast replicative aging experiments that have facilitated the discovery and characterization of these trajectories.

## Materials and reagents

1. 100 mm diameter silicon wafers (University Wafer, catalog number: 452)
2. SU-8 2005 (Kayaku Advanced Materials)
3. SU-8 2010 (Kayaku Advanced Materials)
4. SU-8 2015 (optional) (Kayaku Advanced Materials)
5. SU-8 Developer (Kayaku Advanced Materials)
6. Isopropanol (e.g., Spectrum Chemical, catalog number: 67-63-0)
7. Acetone (e.g., Spectrum Chemical, catalog number: 67-64-1)
8. Ethanol (e.g., Koptec, catalog number: 64-17-5)
9. Methanol (e.g., Sigma-Aldrich, catalog number: 179337)
10. Heptane (e.g., Sigma-Aldrich, catalog number: 494526)
11. Deionized water (from purification system, e.g., Thermo Scientific, model: 7143)
12. Trichloro(1H,1H,2H,2H-perfluorooctyl)silane (Fisher Scientific, catalog number: 448931-10G)
13. Chrome glass masks, custom-made according to the user’s AutoCAD design file and ordered from a company such as HTA Photomask (https://htaphotomask.com/)
14. Pyrex dish for development (Fisher Scientific, Pyrex, catalog number: 08-741F)
15. Pyrex dish for wafer handling (Fisher Scientific, Pyrex, catalog number: 08-747E)
16. Plastic weighing dishes (e.g., Fisherbrand, Fisher Scientific, catalog number: S67090A)
17. SYLGARD 184 Silicone Elastomer kit (PDMS) (Dow, catalog number: 2646340 or purchase from Fisher Scientific, catalog number: NC9285739)
18. Kapton tape (e.g., Uline, Fisher Scientific, NC0912751)
19. Adhesive labels (e.g., Fisher Scientific, catalog number: 15922)
20. Plastic bottles for aliquoting photoresist (e.g., Thermo Scientific Nalgene, Fisher Scientific, catalog number: 02-925-3C)
21. Microcentrifuge tubes (e.g., Thomas Scientific, Fisher Scientific, catalog number: NC9448938)
22. Scotch Magic tape (3M, catalog number: B0000DH8HQ)
23. Aluminum foil (e.g., Thomas Scientific, catalog number: 1181K86)
24. Single edge razor blades (e.g., Fisher Scientific, Fisherbrand, catalog number: 12-640)
25. 0.5 mm biopsy puncher (World Precision Instruments, catalog number: 504528)
26. Cutting pad (e.g., Qiagen Harris Cutting Mat, catalog number: WB100020)
27. Cover glass slides 45 × 50 mm (Oracle, Brain Research Laboratories, catalog number: 4550-1.5D)
28. Glass stir rod (e.g., Millipore Sigma, catalog number: Z549768)
29. Timer (e.g., Fisher Scientific, Fisherbrand, catalog number: 14-649-17)

## Equipment

1. Wafer tweezers (e.g., Electron Microscopy Sciences Rubis Style 39S-4, Fisher Scientific, catalog number: 50-239-33)
2. Tweezers (e.g., for an assorted set, Kaisi, catalog number: B07GLJ7627)
3. Scissors (any generic brand, e.g., Fisher Scientific, catalog number: S173182)
4. Spin coater (e.g., Laurell Technologies Corporation, model: WS-650S-6NPP/UD2)
5. Wafer alignment tool (Laurell Technologies Corporation)
6. Mask aligner (e.g., EV Group, model: EVG620 Lithography/NIL System)
7. Hot plates (e.g., Wenesco)
8. Nitrogen spray gun (supplied in cleanroom facilities, e.g., Terra Universal, catalog number: 2002-21)
9. Profilometer (e.g., Veeco, Dektak 150)
10. Upright microscope (e.g., Zeiss, Axio Imager)
11. Vacuum desiccator (e.g., Nalgene, Thermo Fisher Scientific, catalog number: 5310-0250)
12. Stereomicroscope (e.g., Amscope, catalog number: SM-4B)
13. UVO-Cleaner (Jelight, model: 42)
14. Oven (e.g., Fisher Scientific, catalog number: 13246516GAQ)

## Software

1. AutoCAD from AutoDesk (https://www.autodesk.com/products/autocad). Requires license but this is free to download for students and educators.

## Procedure

- **Obtain chrome glass masks for photolithography**
  1. As part of this protocol, we have created an AutoCAD file with only the designs for the microfluidic devices used in Paxman et al. (2022) and included it in the Supplement. See Figure 1 for the design of the microfluidic device. Email the .dwg AutoCAD file to a company such as HTA photomask, which makes chrome glass masks. Order two 5 × 5 inch chrome photomasks made of quartz glass. Use ± 0.25 μm tolerance for the first mask containing the cell traps and ± 0.5 μm tolerance for the second mask containing the media channels. Have the closed objects on the .dwg file printed as clear on the mask and have the printing as *right reading down* with the chrome side facing down.
    **Figure 1.**
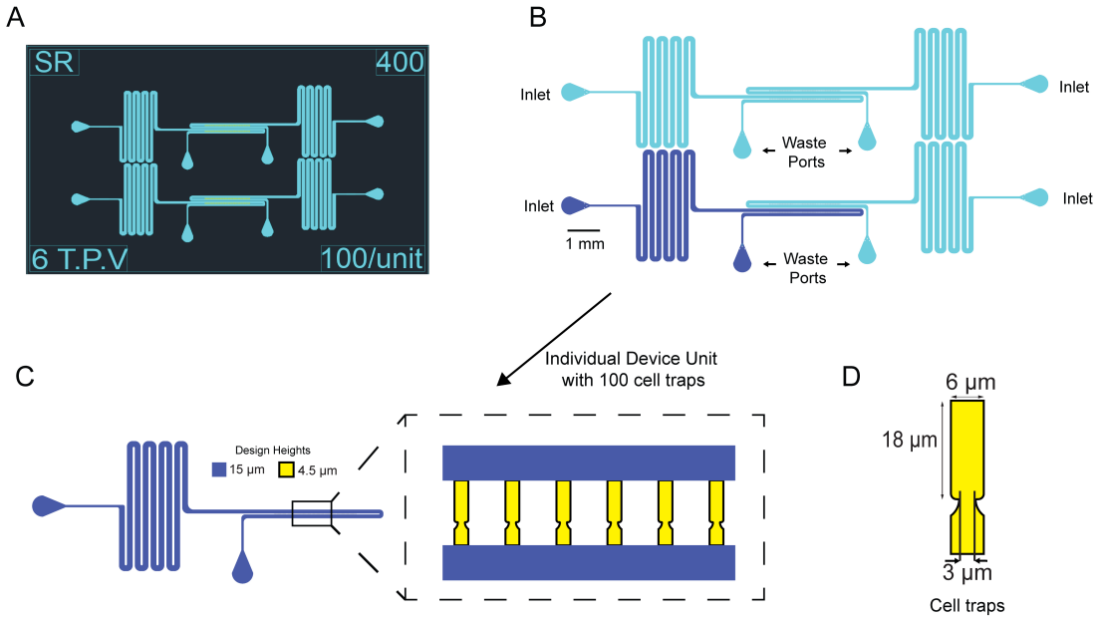
Layout and design of yeast aging microfluidic device. (A) Screenshot of the AutoCAD design file of the device. (B) Schematic of device layout with the inlet ports and outlet/waste ports labeled. The inlet ports are where cells are loaded into the device and afterward a fresh syringe with media is connected. Four units comprise a single device. (C) Single unit with 100 cell traps. Cell traps were designed to be 4.5 μm tall and media channels were designed to be 15 μm tall. D. Dimensions of cell traps.
- **Prepare the wafer for photolithography**
  1. Cleaning the wafer (optional):
    - Sonicate the wafer in acetone for 15 min.
    - Rinse wafer with methanol and then sonicate in methanol for 5 min.
    - Rinse wafer with isopropanol and then sonicate in isopropanol for 5 min.
    - Rinse wafer with deionized water.
*Note: While this step may be useful for certain kinds of silicon wafers, we have found it to be unnecessary if using the wafers included in the Materials and Reagents section (100 mm wafers). Therefore, if using these wafers, we recommend skipping this step.*
  2. Dehydration bake:
    - Bake the wafer at 150 °C for 15 min followed by a 15 min cool down to room temperature (RT).
- **Photolithography for cell trapping layer (Layer 1)**
  1. See Figure 2 for an overview of the process for Layer 1.
    **Figure 2.**
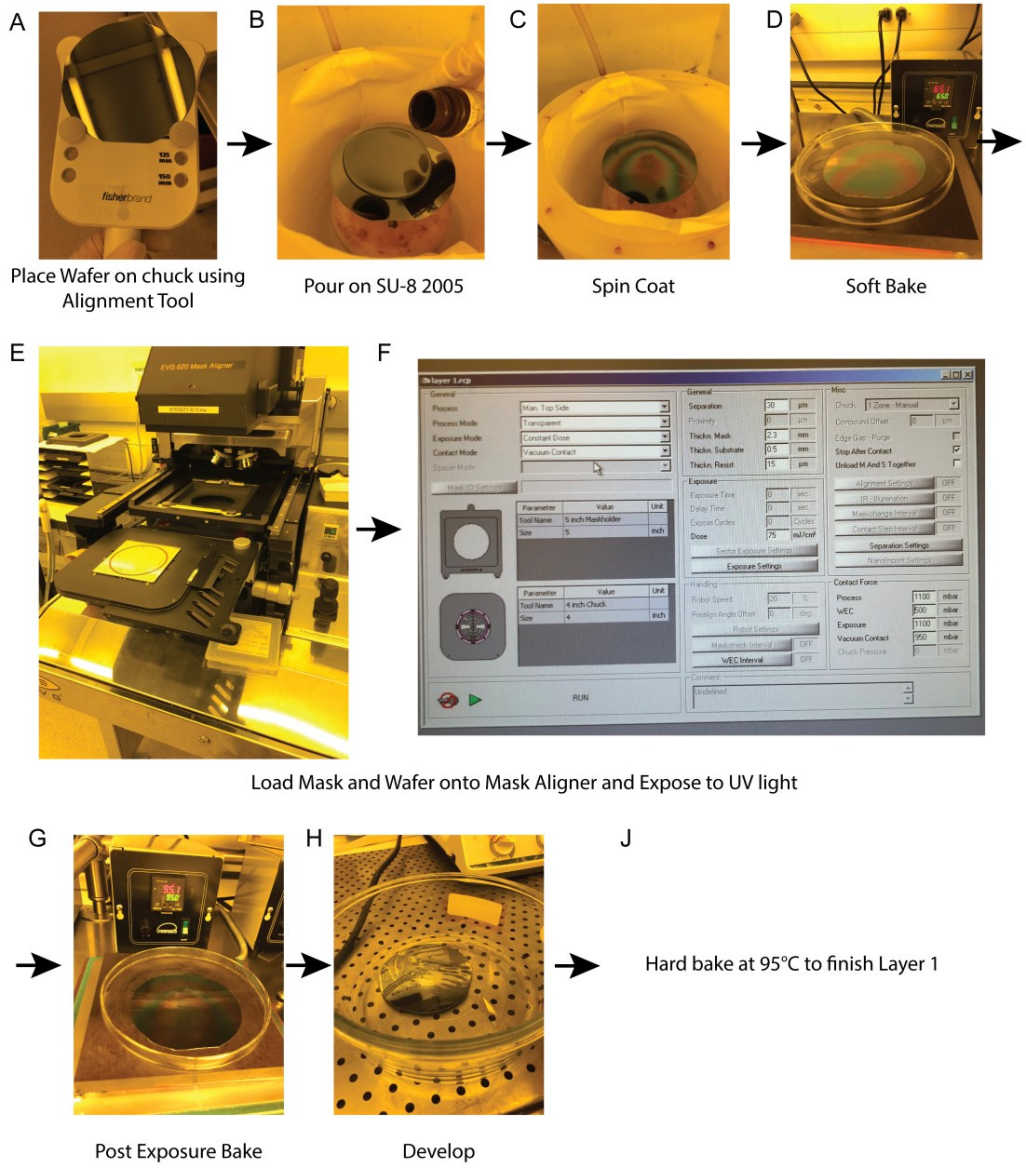
Overview of steps for photolithography process of Layer 1 for the cell trapping layer. (A) The wafer is placed on the spinner chuck using the alignment tool. This ensures that the wafer is centered on chuck. (B) SU-8 2005 is poured on the wafer, covering its entire surface. (C) Spin coating of the wafer with photoresist. (D) Soft bake of the wafer. (E) The mask is loaded onto the EVG620 lithography system as shown, followed by the wafer. (F) Recipe used on the EVG620 lithography system showing important parameters for the process for Layer 1, including the use of vacuum contact and an exposure dose of 75 mJ/cm^2^. (G) Post-exposure bake of the wafer. (H) Development of the wafer in SU-8 Developer to remove photoresist that has not been crosslinked via ultraviolet (UV) light exposure. (J) To complete the process, the wafer is hard baked at 95 °C for 5 min.
  2. Lift the spin coater lid and use the wafer alignment tool to place the wafer on the center of the chuck.
  3. Flood the wafer with SU-8 2005 photoresist so that the entire wafer is covered in resist.
  4. Set the time on the spin coater to 40 s and set the spin speed to 3,000 rpm. For the acceleration, select the preset value that is closest to 3,000 rpm/s, so that the wafer will reach its desired spin speed in 1 s.
*Note: We note that these spin parameters are unconventional, as data sheets for SU-8 2000 series photoresists recommend a first step at a speed of 500 rpm and acceleration of 100 rpm/s. However, since the target height of the cell traps is 4.5 μm, we found it more reliable to achieve this height by omitting this first step and spinning immediately at the high speeds than by following the recommended protocol from the manufacturer, in which we found it difficult to reliably spin this photoresist down to less than 5 μm. Therefore, in our experience, modulating the initial acceleration is a powerful method for tuning the photoresist height.*
  5. When spin coating has been completed, soft bake the wafer at 65 °C for 9 min. Place the lid of a Pyrex dish over the wafer while it is on the hot plate. Position the lid so that it slightly hangs over the end of the hotplate. When time is up, remove the Pyrex dish lid and use wafer tweezers to remove the wafer from the hot plate. Place the wafer on the lid and allow it to cool down to RT for 3 min.
*Note: Placing the lid over the wafer is done to give the wafer a surface of equal temperature to cool on, so that it does not cool too fast. This is done for all baking steps in this protocol.*
  6. Expose the wafer to UV light on the EVG620 using the Layer 1 chrome glass mask without a filter in place at 75 mJ/cm^2^ using the vacuum contact setting.
  7. Remove the wafer from the mask aligner.
  8. Perform a 2 min post-exposure bake at 95 °C. Arrange the Pyrex dish lid over the wafer as before. Allow 3 min to cool down to RT.
  9. Pour SU-8 Developer in a large Pyrex dish and develop the wafer for 2 min. During this time, gently shake the dish back and forth and side to side. After 2 min, take the wafer out of the dish and rinse it with fresh developer for ~10 s; then, rinse with isopropanol for ~20 s. Blow the wafer dry using a nitrogen spray gun.
  10. Hard bake the wafer at 95 °C for 5 min with a 3 min cool down to RT afterward.
  11. Measure the height of the layer using a profilometer and assess feature integrity under a microscope. While there is inherent variability in the spin coating process, it is important to verify that the measured height is close to that of the design.
*Note: When assessing the first layer height and feature integrity under an upright microscope, ensure that trap dimensions are close to those shown in Figure 1C and 1D and that there are no cracks or dents in the photoresist layer. Successfully built wafers should have dimensions within ±10% of the design specifications for the first layer.*
- **Photolithography for media channel layer (Layer 2)**
  1. Tape over the alignment markers on the wafer (Figure 3). To do this, cut out a piece of Kapton tape and cut out a small square from the adhesive labels that is large enough the cover the alignment markers. Using tweezers, place the sticky side of the cut-out label square onto the sticky side of the Kapton tape. Place this over the alignment markers on the wafer so that the non-sticky side of the label is covering the alignment markers. Use a tweezer to press down on the tape surrounding the alignment markers so that it sticks to the wafer. Trim Kapton tape hanging off the wafer but leave enough excess to grab with a tweezer (tape will be removed after spin coating).
    **Figure 3.**
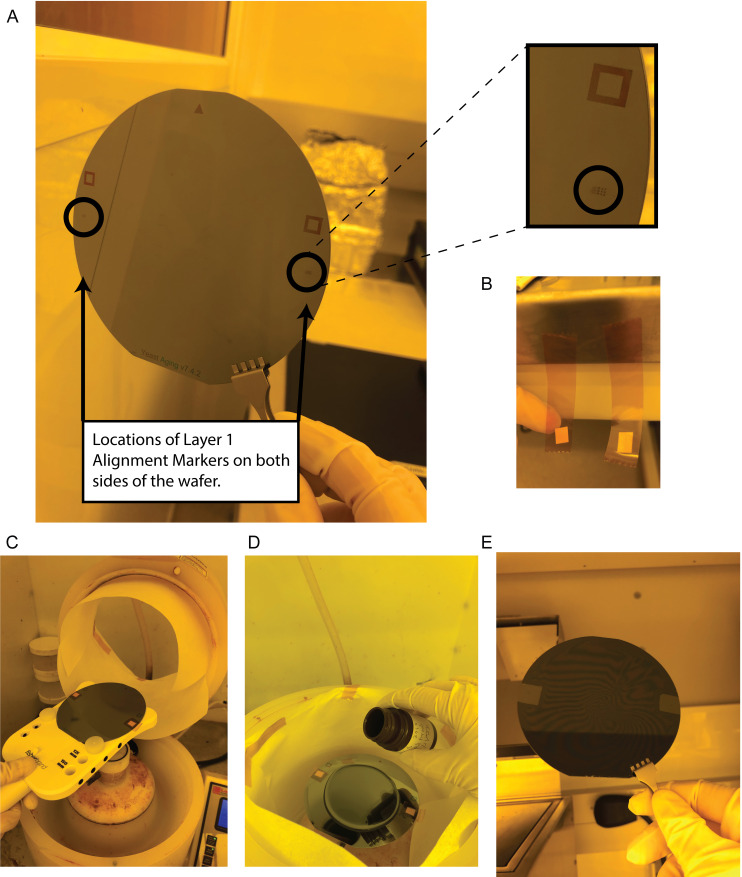
Taping over alignment markers on Layer 1. (A) Location of alignment markers on wafer, including a zoomed in image (top right). (B) Tape setup for covering alignment markers. (C) View of loading wafer onto the spin coater with alignment markers covered with tape. (D) Pouring on second layer photoresist, SU-8 2010. (E) Removal of tape covering alignment markers after spin coating.
  2. Pour approximately 4 mL of SU-8 2010 onto the wafer.
  3. Set the time on the spin coater to 40 s and set the spin speed to 1,450 rpm. For the acceleration, select the preset value that is closest to 1,450 rpm/s, so that the wafer will reach its desired spin speed in 1 s.
*Note: In the original build for this wafer, these settings were used to maintain consistency with the method of spinning the first layer. However, in later builds we have switched to a more standard approach for achieving a 15 μm layer by using SU-8 2015 and spinning in two steps: step 1 is at 500 rpm with a 136 rpm/s acceleration for 10 s, and step 2 at 3,000 rpm with a 272 rpm/s acceleration for 40 s. We recommend this latter approach for the build.*
  4. With a tweezer, carefully remove the tape over the alignment markers on the wafer.
  5. Soft bake the wafer at 65 °C for 15 min with a 3 min cool down to RT afterward.
  6. Alignment of Layer 1 to the mask for Layer 2:
    - See Figure 4 for the design of the alignment markers, the goal of the alignment process, and example images during alignment. Align Layer 1 and Layer 2 alignment markers by adjusting the X, Y, and Theta knobs on the EVG620 mask aligner. The most efficient way to accomplish this is to first tune the focus and position of the lenses on the EVG620 to locate the alignment markers on the Layer 2 mask. Then, turn the knobs to locate the alignment markers on the wafer. Then, to align the two sets of markers, correct half in the Y direction and half in the Theta direction until all sets of squares are fully aligned (see Figure 4). Perform any intermittent corrections in the X direction as needed during this process.
    **Figure 4.**
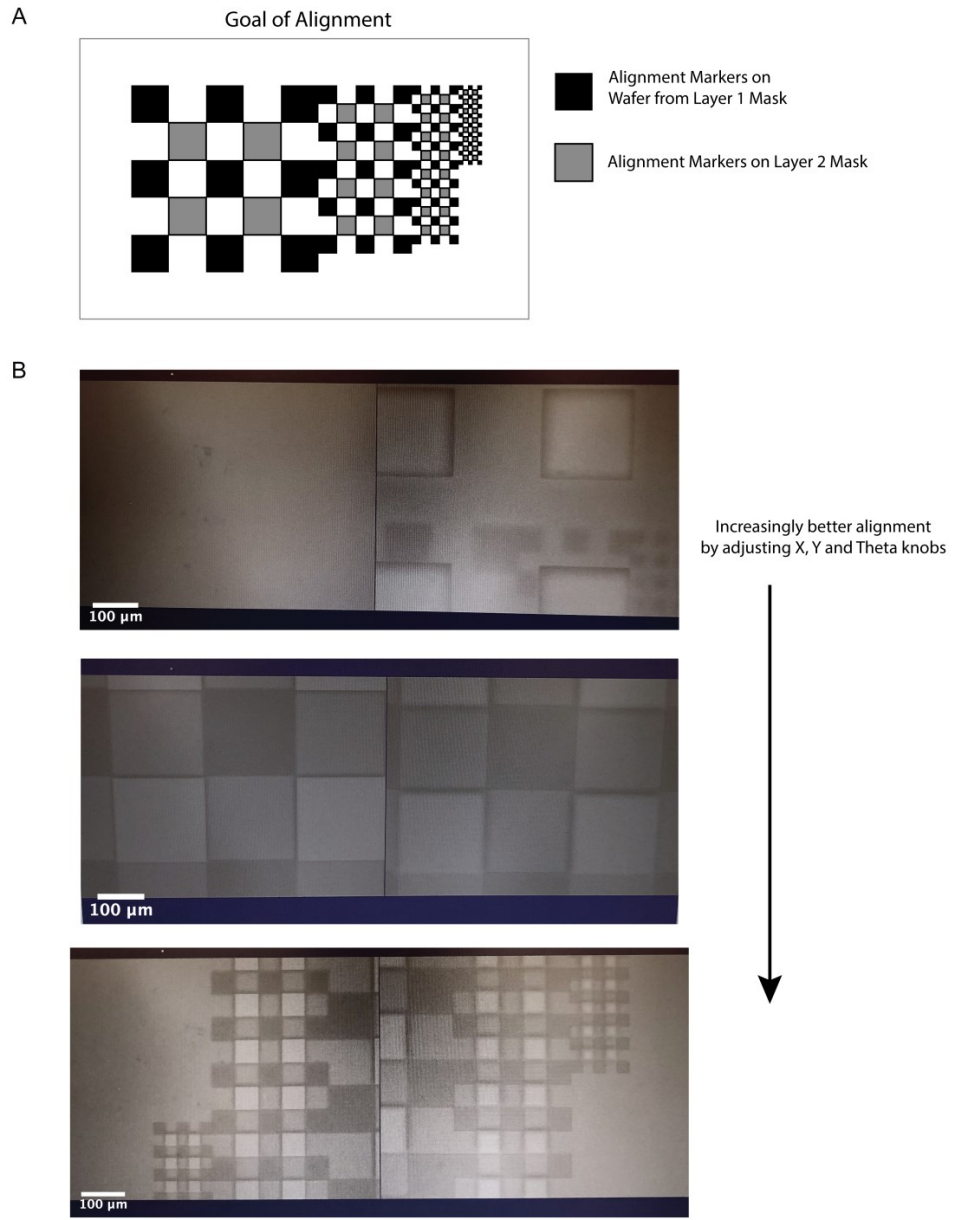
Alignment of Layer 1 and Layer 2. (A) Schematic of the final goal of the alignment process with a single set of alignment markers on the wafer and on the Layer 2 mask shown correctly aligned. (B) Progressing views of alignment process. Markers on the wafer and the mask begin out of alignment (top) but are gradually put into correct alignment by tuning the X, Y, and Theta knobs on the mask aligner. The largest markers are aligned first (middle), followed by further improvement of alignment by correctly orienting the smallest set of markers (bottom), thereby completing the alignment process.
  7. Expose the wafer on the EVG620 using the Layer 2 chrome glass mask without a filter at 125 mJ/cm^2^ using the hard contact setting.
  8. Post-exposure bake at 95 °C for 5 min with a 3 min cool down to RT.
  9. Pour fresh SU-8 Developer into the large Pyrex dish and develop the wafer for approximately 5 min with gentle shaking. Rinse with fresh developer and then isopropanol and blow dry with a nitrogen spray gun.
  10. Hard bake at 95 °C for 5 min with a 3 min cool down to RT.
  11. Measure the height of the media channels with a profilometer and assess feature integrity of the final wafer using an upright microscope (Figure 5A and 5B).
*Note: Ensure that the photoresist layer is free of large cracks and dents when analyzing the wafer under an upright microscope. Error tolerability in height for the second layer is larger than that for the first; however, aim for ±20% from the design specifications. Importantly, make sure the traps are well aligned between the channels as in Figure 5B.*
    **Figure 5.**
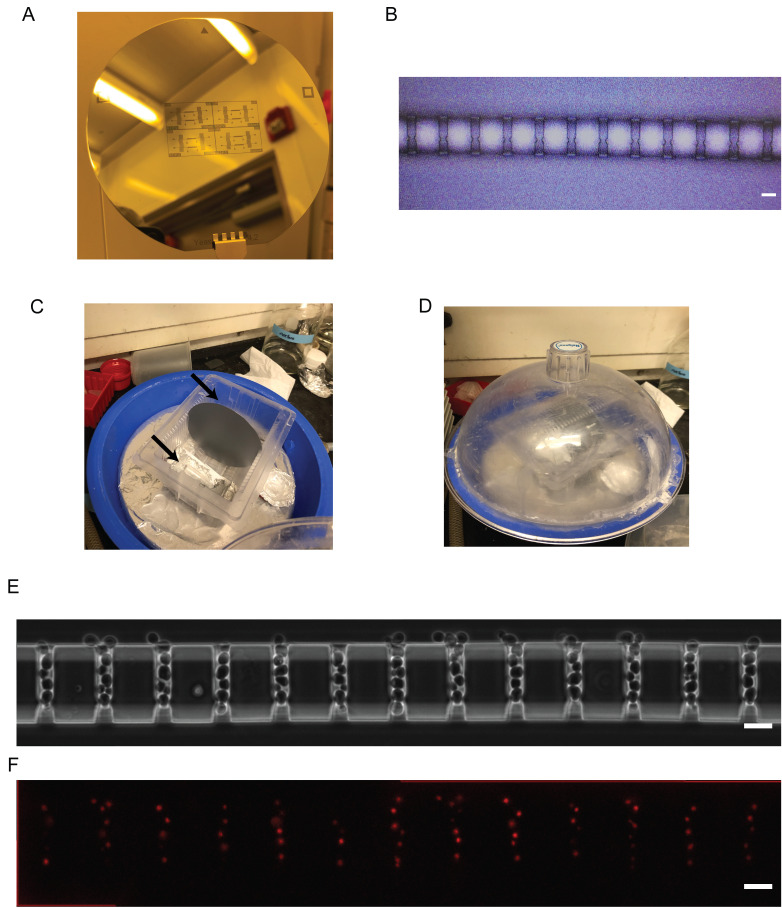
Final wafer, silanization, and application of the polydimethylsiloxane (PDMS) microfluidic device. (A) Fully constructed wafer. (B) Check the alignment between the two layers of the final wafer under a microscope. (C) Wafer placed in the silanization chamber with two Eppendorf tubes, each containing 20 μL of silane placed on either side. Black arrows show the placement of the tubes. (D) Wafer in the closed silanization chamber with the vacuum pump turned on. (E) Phase contrast image from Paxman et al. (2022) of cells growing in the PDMS microfluidic device (scale bar = 10 μm). (F) Fluorescence image from Paxman et al. (2022) of cells with a nuclear iRFP marker growing in the PDMS microfluidic device (scale bar = 10 μm).
- **Wafer silanization**
  1. In a chemical fume hood, place the wafer in a vacuum desiccator and place in two microcentrifuge tubes. Pipette 20 μL of trichloro(1H,1H,2H,2H-perfluorooctyl)silane into each tube (Figure 5C). Start the vacuum and allow the wafer to be exposed to the silane for 7 min (Figure 5D). For more details on our vacuum desiccator setup, see Ferry et al. (2011). **Caution:** Silane is toxic, and this step must be done within a chemical fume hood with appropriate personal protective equipment.
- **Soft lithography using PDMS**
  1. Weigh out 30 g of SYLGARD 184 Base; then, in the same plastic weighing dish, add 3 g of SYLGARD 184 curing agent. Thoroughly mix together with a glass stir rod.
  2. Place PDMS mixture in a vacuum desiccator for approximately 30 min to remove bubbles.
  3. Wrap the wafer in aluminum foil so that the aluminum foil forms a bowl around the wafer and the wafer is sitting flat.
  4. Pour PDMS onto the wafer.
  5. Place the wafer back in the vacuum chamber for approximately 30 min or until all bubbles have been cleared.
  6. Bake the PDMS at 80–90 °C for at least 1 h (overnight optional).
  7. Use a razor blade to remove aluminum foil from the back of the wafer. Gently peel up the aluminum foil and PDMS on the flat edge of the wafer and peel off the PDMS.
  8. Cut out individual devices with a razor blade.
  9. Use a 0.5 mm biopsy puncher to punch the inlet and outlet ports on each device under a stereomicroscope.
- **Bonding PDMS devices to glass coverslips**
  1. Rinse PDMS devices with ethanol and then deionized water. Blow dry with a nitrogen or air spray gun.
  2. Clean the PDMS devices by placing a piece of Scotch tape on the feature side of the PDMS device and using the blunt end of a tweezer to go over the tape in a back-and-forth motion. Apply enough pressure to ensure that the tape works its way into the features and withdraws any dust. Repeat this process at least four times on the feature side and at least once on the non-feature side.
  3. Clean glass coverslips with heptane. Rinse with methanol and then with deionized water. Blow dry with a nitrogen or air spray gun.
  4. Turn on the UVO-Cleaner and run it for 5 min without any samples in.
  5. Place the cleaned glass slides and PDMS devices, feature side facing up, into the UVO-Cleaner and run for 3 min.
  6. Flip PDMS devices over and place the feature sides of the devices in contact with the glass slides.
  7. Place devices in an 80–90 °C oven overnight to bond. After this point, devices should be used for experiments within two weeks [see Paxman et al. (2022) for details on experimental setup]. As a reference, yeast cells growing in the device can be seen in Figure 5E and 5F.

## Validation of protocol

Paxman et al. (2022). Age-dependent aggregation of ribosomal RNA-binding proteins links deterioration in chromatin stability with challenges to proteostasis. eLife (Figure 1, panels A–D; Figure 3, panels A–C; Figure 4, panels A–D; Figure 5, panels A and B; Figure 6, panel B).

## Supplementary information

The following supporting information can be downloaded here:

The AutoCAD file with the designs for the microfluidic devices used in Paxman et al. (2022).
